# End-Systolic Eccentricity Index Obtained by Enhanced Computed Tomography Is a Predictor of Pulmonary Vascular Resistance in Patients with Chronic Thromboembolic Pulmonary Hypertension

**DOI:** 10.3390/life12040593

**Published:** 2022-04-17

**Authors:** Yoshinori Tsutsumi, Shiro Adachi, Yoshihisa Nakano, Shingo Iwano, Shinji Abe, Katsuhiko Kato, Shinji Naganawa

**Affiliations:** 1 Department of Radiological Technology, Nagoya University Hospital, 65 Tsurumai-cho, Showa-ku, Nagoya 466-8560, Japan; ytsutsumi-ngy@med.nagoya-u.ac.jp (Y.T.); shinji.abe@med.nagoya-u.ac.jp (S.A.); 2 Department of Cardiology, Nagoya University Hospital, 65 Tsurumai-cho, Showa-ku, Nagoya 466-8560, Japan; sadachi@med.nagoya-u.ac.jp; 3 Department of Public Health and Health Systems, Nagoya University Graduate School of Medicine, 65 Tsurumai-cho, Showa-ku, Nagoya 466-8560, Japan; middlefield0608@med.nagoya-u.ac.jp; 4 Department of Radiology, Nagoya University Graduate School of Medicine, 65 Tsurumai-cho, Showa-ku, Nagoya 466-8560, Japan; naganawa@med.nagoya-u.ac.jp; 5 Functional Medical Imaging, Biomedical Imaging Sciences, Division of Advanced Information Health Sciences, Department of Integrated Health Sciences, Nagoya University Graduate School of Medicine, 1-20, Daikominami 1-chome, Higashi-ku, Nagoya 461-8673, Japan; katokt@met.nagoya-u.ac.jp

**Keywords:** chronic thromboembolic pulmonary hypertension, computed tomography, biventricular function, pulmonary angiography

## Abstract

The usefulness of the parameters of biventricular function simultaneously measured using enhanced multi-detector computed tomography (MDCT) pulmonary angiography in patients with chronic thromboembolic pulmonary hypertension (CTEPH) has not been clarified. This study aimed to verify the correlation between left and right ventricular (RV) parameters and pulmonary vascular resistance (PVR). Patients who underwent enhanced MDCT before diagnostic right heart catheterization at Nagoya University Hospital between October 2014 and April 2021 were enrolled. The correlation of biventricular function and volume parameters with PVR was assessed. Eighty patients were retrospectively analyzed. Patients’ mean age was 65 ± 13 years, mean PVR was 9.1 (range, 6.1–11.3) Wood units, and mean end-systolic eccentricity index (esEI) was 1.76 ± 0.50. RV end-systolic volume (ESV) (*p* = 0.007), RV cardiac output (CO) (*p* < 0.001), RV ejection fraction (*p* < 0.001), LV end-diastolic volume (EDV) (*p* < 0.001), left ventricular (LV) ESV (*p* = 0.006), LVCO (*p* < 0.001), end-diastolic EI (*p* < 0.001), and esEI (*p* < 0.001) were significantly correlated with PVR. The LVEDV (*p* = 0.001) and esEI (*p* < 0.009) were independent predictors of PVR. Systolic pulmonary arterial pressure (PAP) (*p* < 0.001), diastolic PAP (*p* < 0.001), mean PAP (*p* < 0.001), right atrial pressure (*p* < 0.023), and PVR (*p* < 0.001) were significantly higher in the high esEI group than in the low esEI group. The esEI was a simple predictor of CTEPH severity.

## 1. Introduction

Chronic thromboembolic pulmonary hypertension (CTEPH) is a phenotype leading to pulmonary hypertension (PH) and is defined as group 4 according to the Nice clinical classification [1]. As the development of CTEPH has been suggested to be associated with acute pulmonary embolism due to deep vein thrombosis [2], in situ thrombosis in the pulmonary artery (PA), and multiple organized thrombi, a proper term of anti-coagulant therapy is needed. The cumulative incidence of CTEPH has been reported to be 0.1–9.1% within the first two years after symptomatic pulmonary embolism [3], and the annual incidence of CTEPH was suggested to be five individuals per million population per year [4]. Right heart failure is a major direct cause of death in patients with CTEPH who do not fulfil the criteria for pulmonary endarterectomy [5].

The prognosis of patients with CTEPH who do not have an indication for pulmonary endarterectomy has been very poor so far [6]; however, PA dilators and balloon pulmonary angioplasty, or a combination of both treatments dramatically improved prognosis [7]. Owing to this progress in treatment, thrombus detection is essential in the subsegmental branch (≤2 mm), segmental branch, and main PA. As we reported with regard to the usefulness of the refined computed tomography (CT) protocol for the detection of thrombus in the subsegmental artery [8], there are no modalities except for enhanced multi-detector CT (MDCT) to detect thrombus in the PA non-invasively. Progress in MDCT is expected in this field.

Right ventricular (RV) function is a predictor of prognosis in patients with PH [1,9,10,11]. Since estimating RV function is difficult because of the complex anatomy and contraction pattern, volumetric magnetic resonance imaging (MRI) is the gold standard for estimating RV function and has high reproducibility and accuracy. However, its frequent use in daily medical practice is limited by cost and medical resources. Enhanced MDCT is an alternative modality to MRI because RV function as measured by MDCT was very similar to that evaluated with MRI in a meta-analysis [12]. At the same time, evaluation of left ventricular function is also important [13]. In patients with CTEPH, MDCT is essential for surgery to be considered. Biventricular function can be simultaneously measured during enhanced MDCT pulmonary angiography. However, the correlation between biventricular function parameters and the severity of CTEPH has not been clarified. Therefore, this study aimed to verify the correlation between pulmonary vascular resistance (PVR) and the severity of CTEPH and biventricular parameters during MDCT pulmonary angiography.

## 2. Materials and Methods

### 2.1. Ethics Statements

This study was approved by the Human Research Ethics Committee of Nagoya University Hospital (number: 2021-0369).

### 2.2. Study Patients

This single-center, retrospective, observational study was conducted at Nagoya University Hospital in Japan. Consecutive patients who underwent enhanced MDCT before their diagnosis of CTEPH between October 2014 and April 2021 were enrolled in this study. CTEPH was diagnosed using standard diagnostic criteria: a mean pulmonary arterial pressure (mPAP) ≥25 mmHg, pulmonary arterial wedge pressure (PAWP) ≤15 mmHg according to right heart catheterization (RHC), and abnormalities on a ventilation/perfusion scan or residual thrombus of the PA on CT or transcatheter pulmonary angiography after proper term anti-coagulant therapy [1]. Other background diseases of PH were excluded based on physical examination, laboratory test, chest radiography, electrocardiography (ECG), respiratory test, echocardiography, CT, and RHC findings. Finally, eighty patients were enrolled. Patients’ mean age was 65 ± 13 years, and 35% were male (Table 1). Seventy-six percent were classified as World Health Organization (WHO) functional class III or IV. The mean 6-min walking distance (6MWD) was 363 ± 101 m.

### 2.3. Right Heart Catheterization

RHC was performed using a standard protocol under ambient air with a 6-French Swan-Ganz catheter (Edwards Lifesciences, Irvine, CA, USA). The puncture site was the internal jugular vein in all patients under local anesthesia rather than general anesthesia. The transducer was placed at mid-chest level. The PAP, right atrial pressure, RV pressure, PAWP, and cardiac output (CO) were measured. A thermodilution method was used to provide CO. The PVR was calculated as follows: (mean PAP–PAWP)/CO.

### 2.4. Cardiac Computed Tomography Data Acquisition and Reconstruction

ECG-gated cardiac scans were performed using dual-source CT (Somatom Definition Flash, Siemens Healthcare, Forchheim, Germany) with a tube voltage of 120 kV, collimation of 64 × 0.6 mm, and rotation time of 0.28 s/rotation. The helical pitch was adjusted according to the heart rate, and the automatic tube current modulation was set to a quality reference mAs/rotation of 180. The contrast medium was intravenously administered using a dual-head power injector (Dual Shot GX7, Nemoto Kyorindo, Tokyo, Japan), followed by 20 mL of saline at a rate of 4 mL/s. The injection protocol was optimized according to body weight: <40 kg, 80 mL at a rate of 3.3 mL/s, 300-mg iodine/mL iopromide; 40–55 kg, 96 mL at a rate of 4 mL/s, 320-mg iodine/mL ioverso; and ≥55 kg, 96 mL at a rate of 4 mL/s, 370-mg iodine/mL iopamidol. Following the refined CT scan protocol in the pulmonary parenchymal phase using a bolus-tracking method (threshold of 80 HU in the ascending aorta) [8], the ECG-gated cardiac scan was automatically initiated 7 s later without additional injection of the contrast medium. CT images were reconstructed at 10% intervals of the RR interval with a medium soft convolution kernel of I30f (1-mm slice thickness and 0.8-mm increments).

### 2.5. Image Analysis

**Figure 1:**
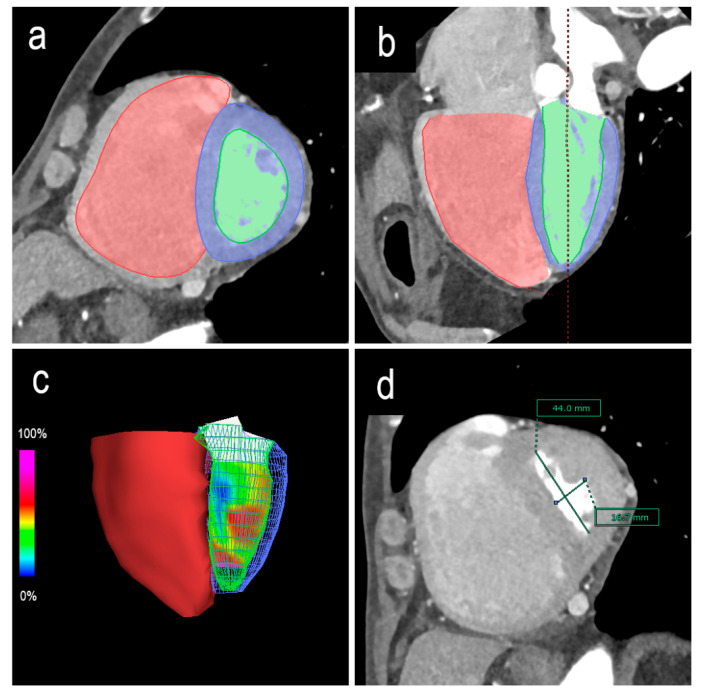
Biventricular end-diastolic and end-systolic volumes and ejection fractions calculated using Simpson’s method. The contours of the ventricular lumens are semi-automatically or manually traced along the short axis (**a**) or long axis (**b**). Three-dimensional reconstruction image of the ventricles (**c**). The eccentricity index is measured on short-axis images at the level of the papillary muscles. In this case, the end-systole eccentricity index (esEI) is the diameter rate of D2/D1 (**d**). D1 = the septal-lateral endocardium distance perpendicular to the septum. D2 = the anterior-inferior endocardium distance parallel to the septum.

Biventricular functions were analyzed using a three-dimensional image analysis application (SYNAPSE VINCENT version 4.3.0003; Fujifilm, Tokyo, Japan). Based on the multiphase reconstruction CT images, end-diastole was defined as 0% of the RR interval, and end-systole was defined as the minimal ventricular volume. The contours of the ventricular lumens at end-diastole and end-systole were semi-automatically or manually traced, and end-diastolic volume (EDV), end-systolic volume (ESV), and ejection fraction (EF) were automatically calculated using Simpson’s method (Figure 1a–c). Since the left ventricle in the end-systolic phase is frequently subjected to morphological displacement, the short-axis centers of the left ventricles were adjusted slice by slice. The papillary muscles were included in the myocardium and excluded from the ventricular volume.

The LV end-diastole index (edEI) and end-systole eccentricity index (esEI) were measured on short-axis images at the level of the papillary muscles as follows: EI = D2/D1 (Figure 1d).

### 2.6. Statistical Analysis

Continuous variables are presented as mean ± standard deviation or as median and interquartile range. Categorical variables are presented as number and percentage.

In the main analysis, the correlation between PVR of RHC and biventricular function parameters of CT was analyzed using Spearman’s rank correlation coefficient. Multivariate analysis was performed using multiple regression analysis. Independent variables of the biventricular function parameters were selected according to their significance in the univariate analysis. The RV ejection fraction was excluded from the multivariate model owing to multicollinearity.

In the sub-analysis, to verify patient characteristics, the esEI was divided into two groups (high esEI group and low esEI group) based on a median of 1.64, and patient characteristics were compared between the two groups. Variables with a normal distribution were analyzed using the independent samples *t*-test, whereas those without a normal distribution were analyzed using Wilcoxon’s signed-rank test. Supplemental data were analyzed using Spearman’s rank correlation coefficient and the multi-regression test. The edEI and RVEDV were excluded from the multivariate model because these parameters were different from the esEI in the cardiac phase.

Statistical analyses were conducted using the SPSS statistical software program (version 24.0 for Windows; IBM Corp., Armonk, NY, USA) or Stata version 17 (Stata Corp., College Station, TX, USA), and a *p*-value < 0.05 was considered statistically significant.

## 3. Results

All patients were treated with warfarin or direct oral anticoagulants, and none of the patients were treated with previous pulmonary vasodilators. The interval from enhanced MDCT to RHC was 26 (range, 7–49) days. Hemodynamic parameters obtained by RHC were as follows: mPAP, 41.2 ± 10.5 mmHg; CO, 3.9 ± 1.0 L/min; and PVR, 9.1 (range, 6.1–11.3) Wood units (Table 2). The biventricular function parameters obtained by enhanced MDCT were as follows: RVEDV, 191.0 (range, 149.7–257.5) mL; RVESV, 132.1 (range, 85.6–188.4) mL; and RVEF, 30.0% (range, 21.9–45.7%) in RV function and LVEDV, 87.0 (range, 71.5–103.0) mL; LVESV, 24.5 (range, 17.9–35.5) mL; and LVEF, 70.0 ± 10.7% in LV function. The edEI and esEI were 1.28 ± 0.15 and 1.76 ± 0.50, respectively. The correlation between PVR and biventricular parameters is shown in Table 3. In univariate analysis, the RVESV (rho = 0.30, *p* = 0.007), RVCO (rho = −0.62, *p* < 0.001), RVEF (rho = −0.57, *p* < 0.001), LVEDV (rho = −0.70, *p* < 0.001), LVESV (rho = −0.31, *p* = 0.006), LVCO (rho = −0.68, *p* < 0.001), edEI (rho = 0.46, *p* < 0.001), and esEI (rho = 0.44, *p* < 0.001) were significantly correlated with PVR. In multi-regression analysis, the LVEDV (*p* = 0.001) and esEI (*p* = 0.009) were independent predictive factors of PVR.

**Figure 2:**
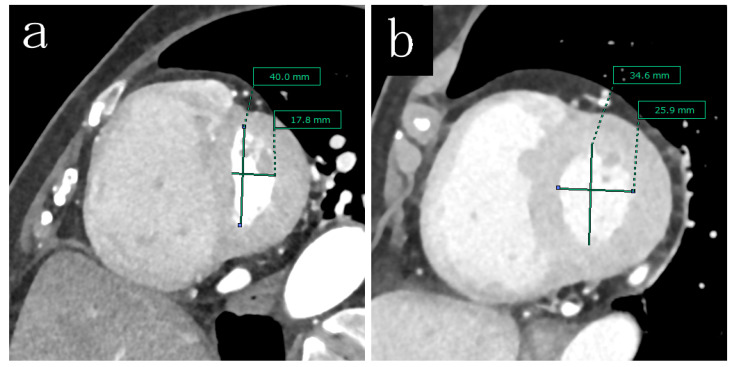
Representative short axis images of the left ventricles in case of different esEI. (**a**) high esEI case, (**b**) low esEI case.

To estimate the factors related to the esEI, the patients’ characteristics were compared between the high and low esEI groups (Table 4). Figure 2 shows the representative short axis images of the left ventricles in case of different esEI. The 6MWD was lower (*p* = 0.017) and brain natriuretic peptide (BNP) levels were higher (*p* < 0.001) in the high esEI group than in the low esEI group. The systolic pulmonary arterial pressure (*p* < 0.001), diastolic pulmonary arterial pressure (*p* < 0.001), mPAP (*p* < 0.001), right atrial pressure (*p* < 0.023), and PVR (*p* < 0.001) were significantly higher in the high esEI group than in the low esEI group. Volumetric-enhanced MDCT revealed that the RVEDV (*p* < 0.001) and RVESV (*p* < 0.001) were larger, whereas LVCO (*p* = 0.003) were smaller in the high-esEI group than in the low-esEI group. The RVEF (*p* < 0.001) and LVEF (*p* = 0.013) were lower in the high esEI group than in the low esEI group. Furthermore, the independent factors related to the esEI were the RVESV (*p* = 0.001), LVCO (*p* = 0.038), and LVEF (*p* = 0.033) in multivariate analysis (Table A1).

## 4. Discussion

This retrospective observational study yielded two main findings: (1) the esEI and LVEDV, both of which were simultaneously measured during diagnostic enhanced MDCT pulmonary angiography, were significantly correlated with PVR; and (2) patients with a high esEI had more severe hemodynamic parameters and biventricular function, and the esEI was significantly correlated with the LVCO, LVEF, and RVESV.

Generally, it is difficult to estimate the severity of PH in patients. Many factors in categorized risk stratification for patients with idiopathic or heritable pulmonary arterial hypertension (PAH), recommended by the European Society of Cardiology, are closely related to RV function, such as BNP levels and CO [1]. Although mPAP is a predictive factor in patients with CTEPH [14], no consensus has been reached regarding the severity of CTEPH. Substantial progress in digital imaging modalities has made it possible to measure RV and LV function easily, and the importance of estimating RV and LV function in patients with CTEPH has been reported [15]. For these reasons, the factor integrating RV function with mPAP, such as PVR, is reasonable [16], and PVR is a predictive factor of survival after pulmonary endarterectomy [17].

Accurate estimation of RV function is difficult because of the complex anatomy of the right ventricle, which is anatomically separated into three segments: inflow, apex, and outflow tract [18]. In addition, the right ventricle is mainly composed of two types of fibers. The superficial layer of the right ventricle is formed predominantly by circumferential muscle fibers, whereas the subendocardial layer is formed by longitudinal muscle fibers. The RV wall motion related to systolic function is organized by the following movements: shortening of the longitudinal axis with traction of the tricuspid annulus towards the apex, radial movement of the RV free wall, which is often referred to as the bellows effect, bulging of the interventricular septum into the right ventricle during LV contraction, and stretching of the free wall over the septum. Therefore, the estimation of RV function using two-dimensional technologies has some limitations. The accuracy and reproducibility of measuring RV function are similar to those of MRI, and enhanced MDCT is an alternative [12]. The disadvantage of enhanced MDCT compared to MRI is radiation exposure, while the advantages are that it is more practical, has a shorter examination time, and is does not require frequent breath holding. Moreover, enhanced MDCT is required to consider suitability for surgical intervention in patients with CTEPH. Measuring biventricular function during MDCT pulmonary angiography simultaneously is considerably beneficial for patients. As CTEPH is a disease that causes dyspnea, it is difficult to hold the breath for a long time and frequently during MRI. Therefore, the more severe the patient, the more difficult it may be to evaluate cardiac function by MRI. In addition, CT pulmonary angiography is necessary for the diagnosis of CTEPH. MDCT was adopted in this study considering another advantage that cardiac function can be evaluated with a single examination.

In the present study, the esEI and LVEDV were significantly correlated with PVR in multivariate analysis. In other words, the severity of CTEPH depends on LV deformity and volume, but not the right ventricle. In terms of compliance, the right ventricle, which has thinner walls than the left ventricle, demonstrates good tolerability to increasing preload^7^. In contrast, it has heightened sensitivity to elevated afterloads, which leads to RV dilation and hypertrophy, and eventually leads to pump failure. When the right ventricle maladapts, RVEF and end-systolic elastance to effective arterial elastance rate decrease, and RVEDV increases, resulting in increasingly more dilation of the right ventricle [18,19]. Prolonged RV shortening and interventricular mechanical asynchrony in addition to an indistensible pericardium develop septum flattering and LV deformity [19], which leads to impaired LV diastolic function and reduced LVCO due to reduced LV preload. The RV and LV are structurally and functionally different; however, they are closely related to ventricular interdependence because of their shared epicardial circumferential myocytes and pericardial space in the right and left ventricles. Therefore, the left ventricle in CTEPH is reflected by not only the extent of RV failure but also the LV status influenced by the dilated right ventricle; therefore, the left ventricle may be an overall functional parameter in CTEPH.

In the present study, the LVEDV and esEI were found to be independent predictors of PVR. Calculating the LVEDV is time-consuming and requires dedicated post-processing software, expert technicians, and time for analysis, whereas the esEI is a very simple and practical parameter that can be easily measured in everyday clinical practice if an ECG-gated cardiac scan is performed. In general, the esEI is measured using echocardiography. The esEI obtained by echocardiography (esEI_ucg_) was correlated with RV pressure [20,21] and is also useful for the diagnosis of RV dysfunction [22]. Furthermore, the esEI_ucg_ was correlated with hemodynamic parameters obtained by RHC and associated with PH outcome measures, such as PH-related hospitalization, medical therapy escalation, and increased BNP levels [23]. The esEI_UCG_ was previously verified in many studies on PH; however, the esEIs measured by MRI (esEI_MRI_) and CT (esEI_CT_) were not adequately verified in PH, especially CTEPH. Although the accuracy of esEI_ucg_ depends on the angle and image quality, there are few dispersions in esEI_MRI_ and esEI_CT_. The esEI_MRI_ predicted PAH with a high sensitivity of 91.9% and was an independent predictive factor of vital prognosis [24]. The esEI_MRI_ was significantly correlated with the mPAP, and the RVEF was negatively correlated with the edEI in patients with congenital heart disease, such as atrial septal defect and tetralogy of Fallot [25]. The esEI_MRI_ was proven to be useful in patients with PAH; however, PVR was not evaluated in either study. The negative correlation between edEI_CT_ and RVEF is consistent with the results of the present study (Table A2). A few reports have verified the usefulness of the esEI_CT_, even though enhanced MDCT is performed to estimate localization of the thrombus and suitability for surgical intervention in patients with CTEPH. Herein, the esEI_CT_ simultaneously measured during MDCT pulmonary angiography was a predictive factor for PVR. This is a great advantage of enhanced MDCT for measuring biventricular function and thrombus localization. It is not necessary to perform MRI to measure ventricular function. Additionally, early estimation of PVR leads to early preparation of pre-interventional pulmonary vasodilators, the so-called pretreatment and treatment for RV failure. The esEI_CT_ was significantly correlated with the LVCO, LVEV, and RVESV. Ventricular interdependence has not yet been clarified. Although the esEI_CT_ is a simple LV deformity index, the LV volume and function as well as the RV volume are influenced by the shared septum. The esEI_CT_ may cast new light on the exploration of ventricular interdependence.

The present study has several limitations. First, the size of the study population was small because patients from only a single PH center at Nagoya University Hospital were enrolled. Therefore, the results of the statistical analysis should be carefully interpreted. Second, the esEI_CT_ was not compared between the esEI_UCG_ and erEI_MRI_ groups. The comparison of accuracy and reproducibility between echocardiography, MRI, and CT is a future task. Third, although enhanced MDCT was performed at the maximal respiratory level to extend the PA, the extent of maximal inspiration could be biased because venous return and the RV volume were reduced during inspiration. Fourth, there was an interval between the enhanced MDCT and diagnostic RHC. If the hemodynamic parameters changed during the interval, this might have affected the results. Lastly, inter- and intraobserver biases were not verified because the similarities of accuracy and reproducibility between volumetric enhanced MDCT and MRI have been proven in a meta-analysis.

## 5. Conclusions

CTEPH severity was predicted using the LVEDV and esEI simultaneously measured during enhanced MDCT pulmonary angiography. The esEI was reflected by the LV volume and function, as well as the RV volume. Estimating the LV volume and function is essential in patients with CTEPH. In particular, the esEI_CT_ is a simple predictor and may be an important index for determining whether medical treatment before intervention is needed in patients with CTEPH.

## Figures and Tables

**Table 1:** Patients’ characteristics.

	Variables	*n* or Mean ± SD or Median (25–75%)
Patient characteristics	Age, years	65 ± 13
	male gender, *n* (%)	28 (35)
	BSA, m^2^	1.58 (1.41–1.74)
	WHO-FC (I/II/III/IV), *n*	0/19/58/3
Exercise tolerance	6MWD, m	363 ± 101
Laboratory data	BNP, pg/mL	90.5 (21.2–315)

SD: standard deviation; BSA: body surface area; WHO-FC: World Health Organization functional class; 6MWD: 6-min walking distance; BNP: brain natriuretic peptide.

**Table 2:** Hemodynamic and biventricular parameters (*n* = 80).

	Variables	*n* or Mean ± SD or Median (25–75%)
Right heart catheterization	sPAP, mmHg	70.5 ± 18.4
	dPAP, mmHg	24.1 ± 7.2
	mPAP, mmHg	41.2 ± 10.5
	RAP, mmHg	6.1 ± 3.3
	PAWP, mmHg	8.7 ± 3.4
	RVP, mmHg	68.6 ± 17.9
	CO, L/min	3.9 ± 1.0
	PVR, Wood unit	9.1 (6.1–11.3)
Parameters of enhanced MDCT	RVEDV, mL	191.0 (149.7–257.5)
	RVESV, mL	132.1 (85.6–188.4)
	RVCO, L/min	4.7 ± 1.4
	RVEF, %	30.0 (21.9–45.7)
	LVEDV, mL	87.0 (71.5–103.0)
	LVESV, mL	24.5 (17.9–35.5)
	LVCO, L/min	4.8 ± 1.4
	LVEF, %	70.0 ± 10.7
	HR, bpm	79.0 ± 14.2
	edEI	1.28 ± 0.15
	esEI	1.76 ± 0.50

SD: standard deviation; sPAP: systolic pulmonary arterial pressure; dPAP: diastolic pulmonary arterial pressure: mPAP: mean pulmonary arterial pressure; RAP: right atrial pressure; PAWP: pulmonary artery wedge pressure; RVP: right ventricular pressure; CO: cardiac output; PVR: pulmonary vascular resistance; MDCT: multi-detector computed tomography; RVEDV: right ventricular end-diastolic volume; RVESV: right ventricular end-systolic volume; RVCO: right ventricular cardiac output; RVEF: right ventricular ejection fraction; LVEDV: left ventricular end-diastolic volume; LVESV: left ventricular end-systolic volume; LVCO: left ventricular cardiac output; LVEF: left ventricular ejection fraction; HR: heart rate: edEI: end-diastolic eccentricity index; esEI: end-systolic eccentricity index.

**Table 3:** Correlation between pulmonary vascular resistance and biventricular parameters (*n* = 80).

	Univariate Analysis			Multivariate Analysis		
Variables		rho	*p* Value	t	95% CI	*p* Value
RV	RVEDV	0.12	0.299			
	RVESV	0.30	0.007	−1.60	−0.030–0.003	0.114
	RVCO	−0.62	<0.001	−1.33	−1.282–0.258	0.189
	RVEF	−0.57	<0.001			
LV	LVEDV	−0.70	<0.001	−2.64	−0.155–(−0.022)	0.010
	LVESV	−0.31	0.006	0.70	−0.072–0.149	0.488
	LVCO	−0.68	<0.001	−1.11	−1.700–0.483	0.271
	LVEF	−0.09	0.435			
	edEI	0.46	<0.001	0.12	−6.037–6.835	0.902
	esEI	0.44	<0.001	2.67	0.712–4.939	0.009

CI: confidence interval; RV: right ventricle; RVEDV: right ventricular end-diastolic volume; RVESV: right ventricular end-systolic volume; RVCO: right ventricular cardiac output; RVEF: right ventricular ejection fraction; LV: left ventricle; LVEDV: left ventricular end-diastolic volume; LVESV: left ventricular end-systolic volume; LVCO: left ventricular cardiac output; LVEF: left ventricular ejection fraction; edEI: end-diastolic eccentricity index; esEI: end-systolic eccentricity index.

**Table 4:** Differences in patients’ characteristics and hemodynamic and biventricular parameters between the high esEI and low esEI groups.

Variables	High esEI (*n* = 40)	Low esEI (*n* = 40)	*p* Value
age, years	62 ± 15	67 ± 11	0.065
6MWD, m	333 ± 106	394 ± 87	0.017
BNP, pg/mL	236 (89–424)	23 (12–95)	<0.001
sPAP, mmHg	79.1 ± 16.7	61.9 ± 15.9	<0.001
dPAP, mmHg	27.3 ± 6.9	20.9 ± 6.1	<0.001
mPAP, mmHg	46.1 ± 9.7	36.0 ± 8.6	<0.001
PAWP, mmHg	9.0 ± 3.5	8.4 ± 3.4	0.456
RAP, mmHg	7.0 ± 3.9	5.2 ± 2.4	0.023
CO, L/min	3.80 ± 1.12	3.96 ± 0.97	0.301
PVR, Wood unit	10.9 ± 4.6	7.5 ± 4.0	<0.001
RVEDV, mL	239.6 ± 59.9	165.5 ± 42.9	<0.001
RVESV, mL	179.1 ± 60.9	101.2 ± 46.7	<0.001
RVCO, L/min	4.62 ± 1.51	5.03 ± 1.23	0.177
RVEF, %	26.7 ± 11.2	41.3 ± 14.8	<0.001
LVEDV, mL	85.8 ± 25.5	92.0 ± 26.1	0.280
LVESV, mL	28.7 ± 12.5	25.4 ± 14.0	0.267
LVCO, L/min	4.34 ± 1.26	5.21 ± 1.32	0.003
LVEF, %	67.0 ± 9.2	72.9 ± 11.4	0.013

esEI: end-systolic eccentricity index; 6MWD; 6-min walking distance; BNP: brain natriuretic peptide; sPAP: systolic pulmonary arterial pressure; dPAP: diastolic pulmonary arterial pressure; mPAP: mean pulmonary arterial pressure; PAWP: pulmonary artery wedge pressure; RAP: right atrial pressure; CO: cardiac output; PVR: pulmonary vascular resistance; RVEDV: right ventricular end-diastolic volume; RVESV: right ventricular end-systolic volume; RVCO: right ventricular cardiac output; RVEF: right ventricular ejection fraction; LVEDV: left ventricular end-diastolic volume; LVESV: left ventricular end-systolic volume; LVCO: left ventricular cardiac output; LVEF: left ventricular ejection fraction.

## Data Availability

Data sharing is not applicable to this article.

## Appendix A

**Table A1:** Correlation between the esEI and biventricular parameters (*n* = 80).

	Univariate Analysis			Multivariate Analysis		
Variables		rho	*p* Value	t	95% CI	*p* Value
RV	RVEDV	0.66	<0.001			
	RVESV	0.70	<0.001	3.51	0.002–0.008	0.001
	RVCO	−0.23	0.036	0.76	−0.070–0.156	0.450
	RVEF	−0.60	<0.001	0.06	−0.167–0.016	0.953
LV	LVEDV	0.16	0.160			
	LVESV	0.18	0.119			
	LVCO	−0.37	<0.001	−2.11	−0.205–(−0.006)	0.038
	LVEF	−0.36	0.001	2.17	0.001–0.216	0.033
	edEI	0.54	<0.001			

CI: confidence interval; RV: right ventricle; RVEDV: right ventricular end-diastolic volume; RVESV: right ventricular end-systolic volume; RVCO: right ventricular cardiac output; RVEF: right ventricular ejection fraction; LV: left ventricle; LVEDV: left ventricular end-diastolic volume; LVESV: left ventricular end-systolic volume; LVCO: left ventricular cardiac output; LVEF: left ventricular ejection fraction; edEI: end-diastolic eccentricity index; esEI: end-systolic eccentricity index.

**Table A2:** Correlations between the edEI and biventricular parameters (*n* = 80).

	Univariate Analysis			Multivariate Analysis		
Variables		rho	*p* Value	t	95% CI	*p* Value
RV	RVEDV	0.36	<0.001	3.36	0.0004–0.0014	0.001
	RVESV	0.46	<0.001			
	RVCO	−0.40	<0.001	−1.07	−0.0455–0.0139	0.290
	RVEF	−0.53	<0.001			
LV	LVEDV	−0.40	<0.001	−2.13	−0.0034–(−0.0001)	0.037
	LVESV	−0.05	0.66			
	LVCO	−0.46	<0.001	−0.36	−0.0486–0.0339	0.722
	LVEF	−0.23	0.04	−0.10	−0.0037–0.0033	0.919
	esEI	0.54	<0.001			

CI: confidence interval; RV: right ventricle; RVEDV: right ventricular end-diastolic volume; RVESV: right ventricular end-systolic volume; RVCO: right ventricular cardiac output; RVEF: right ventricular ejection fraction; LV: left ventricle; LVEDV: left ventricular end-diastolic volume; LVESV: left ventricular end-systolic volume; LVCO: left ventricular cardiac output; LVEF: left ventricular ejection fraction; edEI: end-diastolic eccentricity index; esEI: end-systolic eccentricity index.
